# Identifying a novel locus for psoriatic arthritis

**DOI:** 10.1093/rheumatology/kev273

**Published:** 2015-08-08

**Authors:** Ashley Budu-Aggrey, John Bowes, Anne Barton

**Affiliations:** ^1^Arthritis Research UK Centre for Genetics and Genomics, The University of Manchester,; ^2^NIHR Manchester Musculoskeletal Biomedical Research Unit, Central Manchester Foundation Trust and University of Manchester, Manchester Academy of Health Sciences and; ^3^The Kellgren Centre for Rheumatology, Central Manchester Foundation Trust, NIHR Manchester Biomedical Research Centre, Manchester, UK

**Keywords:** Psoriatic arthritis, psoriasis, specific risk loci, Immunochip, functional characterization

## Abstract

A number of studies have identified genetic risk loci for PsA, the majority of which also confer risk for psoriasis. The stronger heritability of PsA in comparison with psoriasis suggests that there should be risk loci that are specific for PsA. Identifying such loci could potentially inform therapy development to provide more effective treatments for PsA patients, especially with a considerable proportion being non-responsive to current therapies. Evidence of a PsA-specific locus has been previously found at *HLA-B27* within the MHC region. A recent study has provided evidence of non-HLA risk loci that are specific for PsA at *IL23R, PTPN22* and on chromosome 5q31. Functional characterization of these loci will provide further understanding of the pathways underlying PsA, and enable us to apply genetic findings for patient benefit.

Rheumatology key messages

- Genetics studies in PsA could potentially aid the development of more effective therapies for patients.
- Currently, four genetic loci have been found to increase risk of PsA and not psoriasis.
- Characterization of PsA-specific loci will aid in applying genetic findings to patient benefit.

## Introduction

PsA is an inflammatory arthritis that is associated with psoriasis; it has a prevalence of 0.19% in the UK [1] and ∼14% of psoriasis patients are estimated to develop PsA [2]. Although there is clinical overlap between the two diseases, patients with PsA have been found to have a poorer quality of life compared with patients with psoriasis alone [3]. Treatments administered to PsA patients are similar to those used to treat RA and psoriasis; however, there is a significant proportion of PsA patients who are non-responsive to current treatments. This highlights an unmet clinical need for novel therapeutic targets and effective treatments for PsA. Genetic and functional studies have the potential to inform this process by identifying variants that are enriched in patients and whose gene-target could, in turn, be novel targets for treatment. Studies in RA form the paradigm for this approach because genetic susceptibility loci have been shown to be enriched for drug targets [4]. Therefore, genetic studies of PsA have the potential to inform therapy development. Furthermore, as nearly 40% of PsA patients are either undiagnosed or misdiagnosed [5], the identification of PsA-specific loci could also provide markers for identification of prevalent PsA in psoriasis patients as well as identifying psoriasis patients at higher risk of developing PsA later in life. In this article, we review progress in the field of PsA genetics, focusing in particular on the identification of PsA-specific loci.

## Unmet clinical need

At present, treatments for PsA patients are prescribed according to the severity of disease. Patients suffering from the milder or localized forms of disease are treated with NSAIDs and IA CS injections in order to reduce inflammation [6]. Under the current NICE guidelines, the non-biologic DMARDs (nbDMARDs) MTX, LEF, SSZ, AZA and CSA have been licensed to treat patients with moderate to severe forms of disease. Biologic therapies are prescribed for patients who are non-responsive to nbDMARDs [6]. Biologic therapies include adalimumab, etanercept, golimumab, infliximab and certolizumab pegol and act to block the activity of the proinflammatory cytokine, TNFα. Apart from anti-TNF therapies, another biologic treatment is ustekinumab, which targets the shared p40 subunit of the proinflammatory cytokines IL-12 and IL-23 [7].

**Fig. 1:**
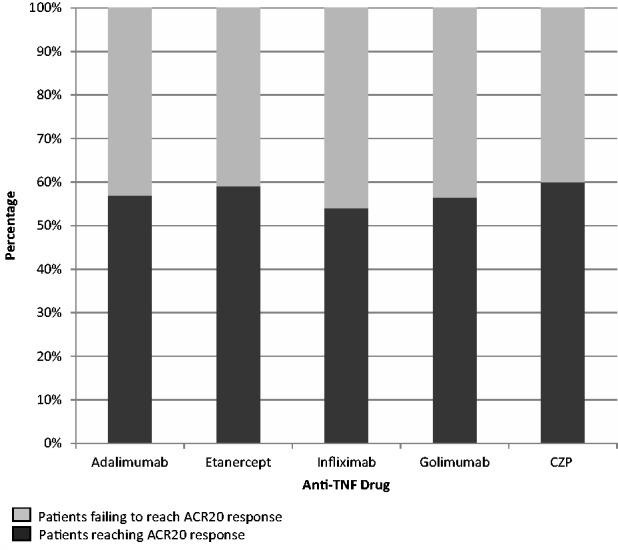
Patients’ response after 24 weeks during clinical trials for adalimumab [12], etanercept [13], infliximab [14], golimumab [15] and certolizumab pegol [16] CZP: certolizumab pegol.

Although a number of therapies are licensed for use in PsA, the effectiveness of existing treatments remains unpredictable and response rates are variable. MTX is most commonly used to treat PsA. However, in a randomized placebo-controlled trial of 221 PsA patients [8] it was reported that MTX failed to significantly improve the inflammatory synovitis experienced by patients. An improvement was observed for skin disease, but it should be noted that the dose of MTX used in that trial was lower than standard practice. The study has also been criticized for the inclusion of patients with mild disease, the use of the PsA response criteria as a primary outcome measure and the study duration [9]. Nonetheless, the results do contradict those of Chandran *et al.* [10], who reported that high doses of MTX can improve the clinical outlook of PsA patients and reduce the progression of joint damage. Furthermore, a study by Eder *et al.* [11] has reported anti-TNFα treatments to be more effective than MTX in preventing progressive radiographic joint damage in patients with erosive PsA. Anti-TNFα therapies have been successful in treating other clinical features experienced by PsA patients, including peripheral arthritis, spondylitis, psoriasis, enthesitis and dactylitis [12–16]. However, the extent of the response reported in trials of PsA is often disappointing, e.g. by week 24, only 52–60% of patients receiving treatment had reached an ACR20 response rate (defined as at least 20% improvement in clinical features) (Fig. 1). This indicates that a notable proportion of patients are non-responsive, with a pattern also seen in RA, for which anti-TNFα treatment is successful in 60–70% of patients [17]. Even fewer PsA patients demonstrate an adequate response to treatment; only 20–30% were reported to reach an ACR70 response rate [12–16]. As the expense of these therapies in the UK can reach up to £10 000 per patient per year, developing more effective therapies would not only provide clinical benefit for patients, but would also benefit the economy.

## Genetic risk loci as therapeutic targets

**Fig. 2:**
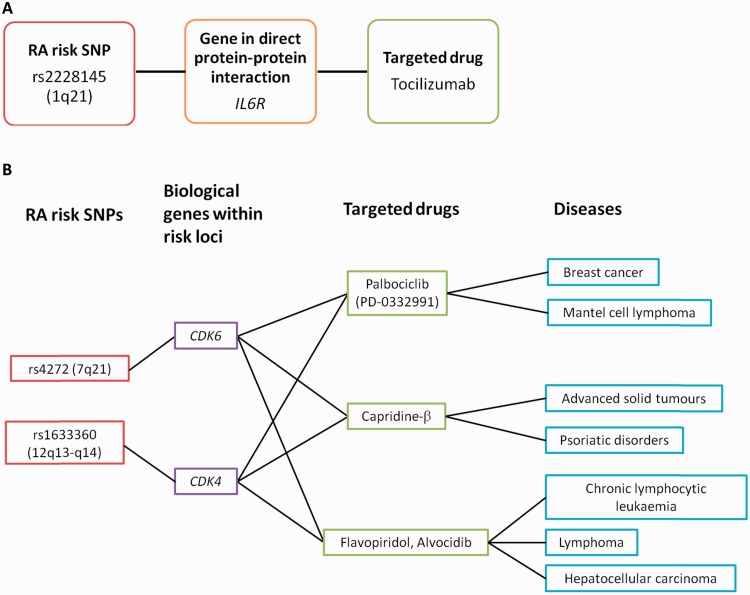
Connection of genetic risk loci with therapeutic targets (**A**) Direct link identified between an RA risk SNP and existing therapeutic target. (**B**) Connections between RA risk loci and therapeutic targets of other diseases. Adapted by permission from Macmillan Publishers Ltd: *Nature* (Okada Y, Wu D, Trynka G *et al.* Genetics of rheumatoid arthritis contributes to biology and drug discovery. *Nature* 2014;506:376–81), © 2014.

The link between genetics and therapeutic targets has been recently demonstrated in RA. A study led by Robert Plenge [4] reported a drug–gene pair with the RA risk gene *IL6R*, whose protein product is the therapeutic target of tocilizumab, an approved drug for RA (Fig. 2A). Overall, an enrichment was found for risk loci for genes that are drug targets for existing RA therapies, serving as a reverse proof-of-principle for the approach. Also in that study, an enrichment of RA risk loci and genes whose protein products are therapeutic targets for existing cancer treatments was reported, highlighting how genetic data can be used to repurpose approved drugs for other diseases (Fig. 2B) [4]. Finally, an example of where genetic knowledge has directly led to a new drug comes from hypercholesterolaemia. This condition is associated with a gain-of-function mutation in proprotein convertase subtilisin/kexin 9 (*PCSK9*), resulting in high levels of low-density lipoprotein cholesterol. The human mAb, REGN727, targets *PCSK9* and has been found to significantly reduce low-density lipoprotein cholesterol levels in individuals with hypercholesterolaemia [18]. These studies demonstrate the role of human genetics in drug target identification and validation, supporting the hypothesis that risk loci can uncover both approved and novel drug targets [4]. This also highlights the potential role that a specific PsA risk locus could play in identifying and validating a novel drug target.

## Genetic loci as predictors of treatment response

Studies in RA have highlighted that genetic susceptibility loci may also predict response to treatment. For example, the major RA susceptibility locus is the *HLA-DRB1* gene. Haplotypes defined by different amino acids at positions 11, 71 and 74 have recently been reported by Viatte *et al.* [19] to be associated with the development of erosions, premature mortality and treatment response to anti-TNF biologic drugs . The identification of novel PsA loci may, therefore, enable better targeting of available therapies to patients—precision medicine. As reviewed by Jani *et al.* [20], studies of genetic polymorphisms predicting response to PsA have highlighted a number of plausible candidates, but require replication in larger case series. Proteomic markers in synovial tissue have also been reported to be correlated with adalimumab response [21]. However, to date, no studies have specifically investigated PsA susceptibility loci to determine whether they also predict treatment response.

## Identifying risk loci

Genome-wide association studies (GWASs) have been highly successful in identifying regions of the genome that confer disease risk, particularly of complex diseases that cannot be explained by Mendelian genetics. The introduction of the Immunochip custom genotyping array (Illumina) provided a means of genotyping known single-nucleotide polymorphisms (SNPs) across confirmed loci from 12 immune-related diseases, with increased power due to the representation of disease-associated loci. Use of the Immunochip has allowed for large regions of association to be refined, resulting in an increase in the number of known susceptibility loci for immune diseases, including RA, psoriasis and Crohn’s disease [22–24]. In psoriasis, this has resulted in the identification of 15 novel risk loci, bringing the total to 36 confirmed loci (*P* < 5 x 10^−^^8^) (Table 1).

**Table 1:** Psoriasis susceptibility loci

SNP	Chromosome	Position (bp)	Combined *P* value	Notable genes
rs2477077	1	197671115	2.4 × 10^−7^	*DENND1B*^a^
rs7552167	1	24518643	8.5 × 10^−12^	*IL28RA*
rs9988642	1	67726104	1.1 × 10^−26^	*IL23R*^b^
rs6677595	1	152590187	2.1 × 10^−33^	*LCE3B*, *LCE3D*
rs11121129	1	8268095	1.7 × 10^−8^	*SLC45A1*, *TNFRSF9*
rs7536201	1	25293084	2.3 × 10^−12^	*RUNX3*
rs62149416	2	61083506	1.8 × 10^−17^	*FLJ16341*, *REL*
rs17716942	2	163260691	3.3 × 10^−18^	*KCNH7*, *IFIH1*
rs10865331	2	62551472	4.7 × 10^−10^	*B3GNT2*
rs27432	5	96119273	1.9 × 10^−20^	*ERAP1*
rs1295685	5	131996445	3.4 × 10^−10^	*IL13*, *IL4*
rs2233278	5	150467189	2.2 × 10^−42^	*TNIP1*^b^
rs12188300	5	158829527	3.2 × 10^−53^	*IL12B*^b^
rs4406273	6	31266090	4.5 × 10^−723^	*HLA-B*, *HLA-C*^b^
rs33980500	6	111913262	4.2 × 10^−45^	*TRAF3IP2*^b^
rs582757	6	138197824	2.2 × 10^−25^	*TNFAIP3*
rs9504361	6	577820	2.1 × 10^−11^	*EXOC2*, *IRF4*
rs2451258	6	159506600	3.4 × 10^−8^	*TAGAP*
rs2700987	7	37386237	4.3 × 10^−9^	*ELMO1*
rs11795343	9	32523737	8.4 × 10^−11^	*DDX58*
rs10979182	9	110817020	2.3 × 10^−8^	*KLF4*
rs1250546	10	81032532	6.8 × 10^−7^	*ZMIZ1*
rs645078	11	64135298	2.2 × 10^−6^	*RPS6KA4*, *PRDX5*
rs4561177	11	109962432	7.7 × 10^−13^	*ZC3H12C*
rs3802826	11	128406438	9.5 × 10^−10^	*ETS1*
rs2066819	12	56750204	5.4 × 10^−17^	*STAT2*, *IL23A*^b^
rs8016947	14	35832666	2.5 × 10^−17^	*NFKBIA*
rs12445568	16	31004812	1.2 × 10^−16^	*PRSS53*, *FBXL19*
rs367569	16	11365500	4.9 × 10^−8^	*PRM3*, *SOCS1*
rs28998802	17	26124908	3.3 × 10^−16^	*NOS2*
rs963986	17	40561579	5.3 × 10^−9^	*PTRF*, *STAT3*, *STAT5A/B*
rs11652075	17	78178893	3.4 × 10^−8^	*CARD14*
rs545979	18	51819750	3.5 × 10^−10^	*POL1*, *STARD6*, *MBD2*
rs34536443	19	10463118	9.1 × 10^−31^	*TYK2*^b^
rs892085	19	10818092	3.0 × 10^−17^	*ILF3*,*CARM1*
rs1056198	20	48556229	1.5 × 10^−14^	*RNF114*
rs4821124	22	21979289	3.8 × 10^−8^	*UBE2L3*

Combined *P*-value—*P*-value from meta-analysis by Tsoi *et al.* [24]. ^a^Psoriasis locus identified by Bowes *et al.* [29]. ^b^Loci associated with PsA at genome-wide levels of significance. Adapted with permission from Macmillan Publishers Ltd: *Nature Genetics* (Tsoi LC, Spain SL, Knight J *et al.* Identification of 15 new psoriasis susceptibility loci highlights the role of innate immunity. *Nat Genet* 2012;44:1341–8), ©2012.

## Genetic studies in PsA

A number of candidate gene studies have been carried out in PsA. Many have focused on variants previously reported to be associated with psoriasis, but only a few loci are confirmed to be associated at accepted thresholds. These include *RUNX3* [25], *IL23A* and *TNIP1* [26]. To date, only one GWAS study has been published that has focused solely on PsA. In a study of 609 German PsA patients with replication in several European cohorts, Huffmeier *et al.* [27] reported variants of *TRAF3IP2* to be significantly associated with PsA, exceeding the genome-wide threshold. Interestingly, the same locus was also identified by a North American consortium, in which PsA cases among a psoriasis cohort were analysed for association [28].

Recently, the largest PsA study to date, which involved the genotyping of 1962 PsA patients and 8923 controls with the Immunochip array, reported seven loci to be associated at a level of genome-wide significance [29] (Table 1). A novel psoriasis risk variant (rs2477077) mapping to *DENND1B* was also found to be associated with psoriasis *per se*. All the previously reported loci with confirmed association with psoriasis were also at least nominally associated with PsA. The overlap of risk loci observed between PsA and psoriasis highlights the strong genetic overlap between the two diseases. However, an Icelandic family study among first-degree relatives with PsA reported a recurrence risk (λ) of 40 [30], while that of psoriasis has been reported to be between 4 and 10 [31]. This difference in heritability suggests that there should be risk loci that are associated specifically with PsA and not with psoriasis.

## Specific PsA risk loci

It has long been recognized that *HLA-B27* is enriched in PsA. In 2012, Eder *et al.* [32] reported *HLA-B27* to be a genetic marker for PsA and not psoriasis, also confirming evidence found in earlier studies [33]. In addition to *HLA-B27*, evidence suggests that other *HLA-B* alleles within the MHC specifically confer risk for PsA. This includes *HLA-B08*, which has also been found to be associated with specific clinical features of PsA [32, 34, 35]. Imputing genes within the MHC region is an approach that has been applied in a number of studies to identify and define associated HLA alleles. This has been demonstrated in psoriasis, for which HLA imputation was carried out with Immunochip and GWAS datasets in order to fine-map risk variants in the MHC region [36]. In doing so, independent effects were found for HLA alleles A, B and C for both PsA and psoriasis [29, 36]. When analysing PsA risk and psoriasis risk separately, the strongest association observed among the HLA variants was with HLA-C*0602, which when conditioned upon revealed an independent contribution from *HLA-B*. This is consistent with the findings reported for PsA by Bowes *et al.* (*P* = 5.85 × 10^−^^52^) [29]. When testing for association of HLA variants with PsA risk versus psoriasis risk, the strongest association was found with HLA-B amino acid position 45, for which HLA–B Glu45 was found to increase PsA risk compared with psoriasis risk. As a result, it was reported that risk heterogeneity between PsA and psoriasis is driven by variants of *HLA-B*. This association differentiates PsA from psoriasis, and as *HLA-B27* and *HLA-B38* also carry Glu at position 45, this explains the stronger association of these alleles with PsA compared with psoriasis [36]. The study demonstrates the use of HLA imputation to highlight the difference in genetic factors that confer risk for PsA and psoriasis, and supports the evidence that there may indeed be additional genetic variants that confer risk specifically for PsA and not psoriasis.

Outside the HLA region, studies have identified a number of loci with larger effect sizes in PsA than psoriasis. This includes variants at *TRAF3IP2*, *REL* and *FBXL19* [27, 37, 38]; however, the loci are associated with both phenotypes and are not specific to PsA.

Evidence has been reported for a specific association with PsA at the *IL13* gene locus. Variants of this gene (rs1800925, rs848 and rs20541) were initially reported to be associated with psoriasis in a study comprising 467 Utah psoriasis patients and 500 controls [39]. The association was replicated in a later, larger study including the same sample set but with additional cases and controls [40]. However, when patients with PsA were excluded from analysis, no association with psoriasis remained [41]. Subsequent studies in Canadian and UK populations [42] confirmed these findings and reported variants of *IL13* to be significantly associated with PsA but not with psoriasis. It is interesting to note that the psoriasis Immunochip study by Tsoi *et al.* [24], which included a larger number of psoriasis cases, did report an association of *IL13* with psoriasis, but cases with known PsA, were not excluded from the analysis. Whether *IL13* represents a PsA-specific association, therefore, requires further investigation.

Recently, a second PsA-specific association has been detected on chromosome 5q31, upstream and independent of the *IL13* association, at the SNP rs715285 (*P* = 2.7 × 10^−^^10^, odds ratio 1.25) [29]. This association was replicated in an independent PsA cohort, but showed only nominally significant association in two independent psoriasis cohorts, suggesting that the association is specific for PsA. In the same study, a specific association was also found with a variant of *IL23R* (rs12044149), independent of a psoriasis-associated variant in the same gene (rs9988642). *IL23* is a drug target for ustekinumab, currently used as a treatment for psoriasis. Although not currently licensed for PsA in the UK, the drug has shown efficacy in treating PsA in clinical trials [43].

Finally, a SNP mapping to *PTPN22* (rs2476601) has recently been reported to be PsA-specific [44]. The SNP is associated with a range of autoimmune diseases, but previous studies in PsA failed to detect consistent evidence for association [45, 46]. However, in a cohort of 3139 PsA cases and 11 078 controls, the association was confirmed at genome-wide levels of significance for the first time (*P* = 1.49 × 10^−^^9^, odds ratio 1.32), with no association detected with psoriasis [44]. As this is also a risk locus for RA [45], one interpretation is that the PsA cohort contained some RA cases with co-incidental psoriasis. Importantly, however, this was addressed using a statistical approach to account for RA susceptibility risk scores.

## Future directions

The studies highlighted demonstrate how dense genotyping and increased study power can aid in the identification of novel susceptibility loci that are specific for PsA. In spite of the benefits of genotyping with the Immunochip array, there are limitations with this approach. As the Immunochip does not provide full genome coverage, there may be susceptibility variants that are not captured. Furthermore, rare variants [minor allele frequency (MAF) < 1%] were not analysed in the current studies. This is important to consider, as rare variants could contribute towards disease susceptibility [47]. One method of detecting disease-associated rare variants is with the use of exome chips, which contain markers identified from exome sequencing, and so are enriched for rare variants [48]. The use of the exome chip has been demonstrated in type 2 diabetes, where the Illumina HumanExome Beadchip was used to genotype 247 870 variants in 8229 non-diabetic males. Novel associations with rare variants in three genes were detected, and the implicated genes were involved with insulin processing and secretion [49]. The application of rare-variant analysis could potentially identify risk loci that are specific for PsA that would otherwise be overlooked with the use of other genotyping platforms.

As the majority of risk variants reported in association studies lie outside gene-coding regions, further investigation is required to identify their role in disease. It has been suggested that such variants contribute towards disease susceptibility by affecting gene expression and regulation [50]. These are known as expression quantitative trait loci (eQTLs), and can be mapped by identifying a correlation between gene expression and genotype at a SNP. The mapping of eQTLs is a useful approach for finding potentially causal genes within a susceptibility locus. Due to the cell type specific activity of most eQTLs and functional variants, it is important that such studies are performed within the relevant cell type for the disease in question [51]. A statistical approach was developed by Trynka *et al.* [51] to identify the most likely cell type responsible for disease causation by investigating the overlap of disease SNPs with the epigenetic mark, trimethylation of histone H3 at lysine 4 (H3K4me3); the hypothesis was that the overlap is cell type specific. Using this approach, the recent Immunochip study found significant enrichment of PsA-associated SNPs and functional elements in CD8^+^ memory primary cells, highlighting this cell type as critical in PsA but not psoriasis.

As well as identifying the most appropriate cell type, it is important to identify the causal variant in order to understand how the variant affects gene function. Candidate SNPs can be prioritized with use of publicly available data from functional genomic experiments. The publication of the Encyclopaedia of DNA Elements (ENCODE) project has provided a resource by which a region of interest can be interrogated for evidence of regulatory function [52]. Functional elements such as transcribed regions, protein-coding regions, transcription factor–binding sites and histone modifications have been successfully mapped across the genome, using approaches including mass spectrometry, chromatin immunoprecipitation followed by sequencing, and RNA sequencing [52]. As well as identifying variants that affect gene expression, ENCODE data can also be used to assess the overlap of risk variants within a functional element. For example, in the recent Immunochip study, publically available data were used to identify eQTLs for 3 of the 20 genes within the 5q31 region [29]. By analysing gene expression in CD8^+^ memory primary cells specifically, the strongest correlation was found with genotype at rs11955347 and expression of *SLC22A5*. This gene encodes a solute carrier and has previously been found to be associated with inflammatory bowel disease; however, further confirmatory work is required before association with this particular gene can be unequivocally established. For example, it is important to consider eQTL studies within stimulated cells, because the function of a cell can differ depending on whether it is active or resting [53]. As demonstrated in a study by Hu et *al*. [53], this could aid in identifying additional eQTLs, as the majority of genes regulated by eQTLs in CD4^+^ T effector memory cells in that study were found in stimulated cells. Methods such as chromosome conformation capture could be applied to identify long-range interactions between a potential causal gene or SNP and regulatory regions located on other chromosomes. Ultimately, the new technique of genome editing using the CRISPR/CAS9 system can be used to validate the causal nature of variants as the system allows allele-specific changes to be introduced into the genetic sequence. This has been demonstrated by Makhlouf et *al.* [54]*,* who found that the binding of transcription factor Yin-Yang has a direct effect on *Xist* transcription in humans and mice.

## Conclusion

Great progress is being made in understanding the genetic basis of PsA. Several tentative conclusions can be drawn from the data so far. First, perhaps unsurprisingly, there appears to be complete genetic overlap between psoriasis and PsA susceptibility loci, with no validated reports of psoriasis loci that are not also associated with PsA, albeit at only nominal significance levels in PsA. Second, there is mounting evidence for an additional genetic contribution to PsA. For some loci, this is a stronger effect size (*TRAF3IP2*, *REL*, *FBXL19*); for others, different genetic variants at the same locus predispose to psoriasis and PsA (*IL23R*) whereas other loci appear specifically associated with PsA (*HLA B27*, 5q31 locus). This not only raises the possibility of targeted screening of the psoriasis population to identify those at higher risk of developing PsA, but also identifies targets for future drug development to improve treatment for this group of patients.
